# Comparison of differences in performance evaluation of faculty by students with faculty’s self-assessment

**Published:** 2014-07

**Authors:** KOUROSH AZIZI, TEAMUR AGHAMOLAEI, NADER PARSA, TAHEREH DABBAGHMANESH

**Affiliations:** 1 Medical entomology department, Health and Nutrition School, Health Sciences Research Centre, Shiraz University of Medical Sciences, Shiraz, Iran;; 2 Public health department, Health School, Hormozgan University of Medical Sciences, Bandar Abbas, Iran;; 3 Cardiovascular Research Center, Shahid Faghihi Hospital, Shiraz University of Medical Sciences, Shiraz, Iran

**Keywords:** Student, Evaluation, Performance, Faculty

## Abstract

**Introduction:** The present study aimed to compare self-assessment forms of coursework taught in the school of public health at undergraduate, graduate, and postgraduate levels and students’ evaluation of the performance of the faculty members at these levels.

**Methods:** The subjects in this cross-sectional study were the faculty members and students of the School of Public Health and Nutrition, Shiraz University of Medical Sciences, Shiraz, Iran. The data were collected using a socio-demographic information form and evaluation forms of professors prepared by the Educational Development Center (EDC). The faculty members were assessed by the students in undergraduate and graduate classes. Among the study subjects, 23 faculty members filled out the self-assessment forms which were then evaluated by 23 students. Then, the data were analyzed using the SPSS statistical 14. Paired t-test was used to compare the students’ evaluation of the faculty members’ performance and the professors’ self-assessment.

**Results:** The mean score of self-assessment of the faculty members who taught undergraduate courses was 289.7±8.3, while that of the students’ evaluation was 281.3±16.1; the difference was statistically significant (t=3.56, p=0.001). Besides, the mean score of the self-assessment of the faculty members who taught graduate courses was 269.0±9.7, while that of the students’ evaluation was 265.7±14.6 but the difference was not statistically significant (t=1.09, p=0.28).

**Conclusions:** Teaching performance perceptions of the faculty were similar to those of the graduate students as compared to the undergraduate ones. This may reflect better understanding of coursework at this level compared to the undergraduate students. Faculty members may need to adjust teaching methods to improve students’ performance and understanding especially in the undergraduate level.

## Introduction

Education is a service which is directly impacted by the provider. Higher education institutions place greater emphasis on meeting the students' expectations and needs. As universities continue to become more student-oriented, students’ perceptions of higher educational facilities and services are becoming more important (1).

Evaluation of coursework and teaching methods are an essential part of the educational process aiming at improving the quality of education. Evaluation by the faculty improves performance in the classroom and quality of the educational experience. The results of evaluations are then incorporated into the relevant coursework. Knowledge of the essential components of quality of educational performance is a necessary part of teaching. The major purpose of evaluation is improvement of educational quality and quantity, collection of data for programming, budgeting financial sources for schools, providing and encouraging the faculty, supporting the faculty’s promotion, improvement of the teaching program, and fulfillment of the students’ learning needs (2). Evaluating the capability, performance, knowledge, and competence of a school's faculty is an important factor for higher education institutions. Thus, senior managers of higher education institutions seek for appropriate methods of evaluation for these important issues (3). Evaluations by students and faculty have been routinely used in academic institutions and have long been an integral part of colleges and universities in driving curricular change and faculty performance (4).

Faculty evaluation is a complex process which contains various interconnected activities and actions, all of which being related to a specific purpose. Without capable, high quality teachers, no education reform effort can possibly succeed. Moreover, without high quality evaluation systems, we cannot know if we have high quality teachers (5).

Evaluation of university faculty members which aims to improve teaching quality is performed through several methods. One of the most common and conflicting methods is evaluation of the faculty by students. This method is commonly used in most universities in spite of controversy over its validity (6). Another method is self-assessments of the faculty (7, 8). There has been debate for decades as to what should be the subject of evaluation of faculty for higher education institutions (9, 10). Although evaluation of students is a necessary part of educational performance, it is not the only and gold standard method to evaluate the role of the faculty. Physical environment, facilities, higher level of managers, and even university personnel should be taken into account in systematic evaluation of the faculty’s training performance (11).

Altman believed that evaluation of the faculty by students could be a key component of educational performance for quality improvement of the training methods (12). He believed it necessary to have a continuous and formative evaluation of the faculty by students.  He suggested that assessment of these results could provide immediate change to improve training methods, learning, and educational performance. Finally, he believed that evaluation should not only be performed at the end of courses.

Another method for evaluation of educational performance is the faculty’s self-assessment. This type of evaluation helps the faculty to gain information on teaching methods, discipline, class control, and level of knowledge. We could propose that self-assessment is the best method of evaluation. When the faculty members evaluate themselves, they are responsible for their own level of performance. Consequently, if there are some deficits in their teaching method, they could correct or improve their own performance (3). Comparison of students’ evaluation and faculty’s self-assessment can lead to better recognition of strengths and weaknesses of teaching. Thus, it can be a part of the faculty’s training performance evaluation (7).

The present study aims to compare evaluation of the faculty members’ educational performance by students and their self-assessment in Health and Nutrition School of Shiraz University of Medical Sciences, Shiraz, Iran. 

## Methods

This cross-sectional research was conducted on 23 faculty members who taught theoretical courses in the first and second semesters of 2013 and the students who took these courses in School of Health and Nutrition, Shiraz University of Medical Sciences. The study data were collected using demographic information questionnaires and evaluation forms of the faculty by undergraduate and graduate students prepared by the Educational Development Center (EDC) for training performance evaluation of the professors of Shiraz University of Medical Sciences. The data included information about the faculty’s self-assessment and evaluation of the faculty by the students who had taken the theoretical courses.

The questionnaire of educational performance evaluation of professors by undergraduate students had 15 items. Each item was responded through a Likert scale and the scores ranged from 0 to 20.  Higher scores showed the professors’ capability and strength, while lower scores represented the professors’ lack of capability and weakness. The maximum score of the questionnaire was 300, indicating the faculty’s excellent performance. The questionnaire of educational performance evaluation of professors by graduate students had 14 items. Each item was responded based on a Likert scale and the scores ranged from 0 to 20. Similar to the previous questionnaire, higher scores represented the professors’ capability and strength, while lower scores represented the professors’ lack of capability and weakness. Besides, the maximum score of the questionnaire was 280, indicating the faculty members’ excellent training method. These questionnaires were approved by the experts in Educational Development Center (EDC) in Shiraz University of Medical Sciences. 

Additionally, the Cronbach’s alpha coefficient of questionnaires of educational performance evaluation of professors by undergraduate and graduate students was between 0.94 and 0.89, which shows the questionnaires’ reliability. 

The faculty first evaluated themselves regarding the coursework they had taught. Also, the professors’ training method was evaluated by the students who had taken the course. In order to control information bias, the faculty’s personal information was removed from the questionnaires and they were given a pin code. In addition, the students filled out the questionnaires anonymously. Before administering the questionnaires, the students and the faculty were informed regarding the study objectives; they were also ensured about their confidentiality.

After all, the data were analyzed using SPSS 14 (SPSS Inc, Chicago, IL, USA) statistical software. Normal distribution of the data was confirmed using Kolmogorov-Smirnov test. Besides, t-test was used to compare evaluation of the faculty by students and professors’ self-assessment. 

## Results

Overall, the data of 23 faculties who had completed self-assessment questionnaires were analyzed.  The faculty who taught courses in undergraduate and graduate levels was evaluated on average by 18 and 5 students, respectively.

Among the professors, 65% were male and 35% were females. Moreover, 17% of the faculty members were instructors, 52% assistant professors, 17% associate professors, and 14% full professors. 

The mean score of the faculty’s self-assessment was 289.7±8.3 in undergraduate coursework performance and 281.3±16.1 by undergraduate students’ evaluation. Consequently, the difference was statistically significant (t=3.56, p=0.001). In addition, the results showed a mild correlation (r≈0.4) between the professors’ self-assessment and undergraduate students’ evaluation. The professors’ and students’ evaluation scores were significantly different regarding 12 out of the 15 questions. Overall, the faculty members’ self-assessment scores were higher than those of the students for all the questions (Table 1).

**Table 1:** Comparison of evaluation of the faculty’s educational performance by undergraduate students and the faculty’s self assessment

**Areas**	**Q.#**	**Question description**	**Self-evaluations**	**Student evaluations**	**P**
			**Mean±SD**	**Mean±SD**	
**Program**	1	Efficient utilization of lecturing time regarding the class schedule material for presentation	19.5±0.62	18.6±1.09	<0.001*
	2	Using educational regulation (Course syllabus, Being on time, Controlling present-Absent of student)	19.4±0.81	18.9±0.85	0.002^*^
	3	Consistency of course materials	19.4±0.69	18.6±1.20	<0.001*
**Teaching skill**	4	Being scientifically knowledgeable and ability of course materials presentation	19.1±1.06	18.5±1.44	0.006^*^
	5	Lecturing capability and delivering main point of course materials	19.2±0.91	18.2±1.60	<0.001*
	6	Delivering important point of course and introducing the necessary, appropriate and useful references	19.1±0.94	18.5±1.40	0.020^*^
	7	Ability of scientific and logical answer to student questions	19.2±0.89	18.2±1.72	<0.001*
	8	Using audio-video or other necessary equipment for training of course materials	19.3±0.76	18.5±1.24	<0.001*
**Evaluation**	9	Identifying method of evaluation at the beginning of semester for student	18.9±1.33	18.4±1.45	0.060
	10	Routine assessment of student progress during the semester and evaluating educational performance feedback	18.3±1.57	18.1±1.37	0.290
**Professional and skillful communication**	11	Applying appropriate ethical, Islamic and professional consideration related to training duties	19.9±0.14	19.3±0.72	<0.001*
	12	Appropriate professional attitude, behavior toward to student	19.8±0.34	19.1±1.33	<0.001*
**Class management skill**	13	Faculty ability regarding class guidance and management	19.8±0.34	19.1±1.33	<0.001*
	14	Faculty ability regarding creative motivation related to scientific issues among student	19.6±0.63	18.7±1.38	0.030^*^
	15	Appropriate learning benefit and educational performance of student from offered coursework	19.3±0.76	18.7±1.38	0.700
**Total scores**			289.7±8.3	281.3±16.1	<0.001*

* Significant level: 0.05

The mean score of the professors' self-assessment was 269.0±9.7 in the graduate level. On the other hand, the mean score of the professors’ evaluation by graduate students was 265.7±14.6. However, no statistically significant difference was found between the two evaluation scores (t=1.09, p=0.28). The results showed a weak correlation (r≈0.1) between the professors' self-assessment scores and graduate students' evaluation scores. 

In this study, a statistically significant difference was observed between the faculty’s self-assessment and students’ evaluation in only 3 out of 14 questions of the questionnaire. The faculty’s self-evaluation was higher than the students’ evaluation in two questions, while the students’ evaluation was higher in the remaining ones (Table 2).

**Table 2:** Comparison of the faculty’s training performance by graduate students and the faculty’s self-assessment

**Areas**	**Q.#**	**Question description**	**Self- evaluations**	**Student evaluations**	**P**
			**Mean±SD**	**Mean±SD**	
**Program**	1	Efficient utilization of lecturing time regarding the class schedule material for presentation	19.3±0.82	19.2±0.74	0.450
	2	Delivering the class lecture according to time schedule	19.5±0.81	19.4±0.73	0.530
	3	Consistency in presentation of course materials without any interruption	19.3±0.67	19.2±0.88	0.350
**Teaching skill**	4	Qualification of lecturer for advanced scientific knowledge related to the relevant course materials presentation	18.9±0.95	18.6±1.69	0.250
	5	Lecturer ability and skill regarding course materials	19.2±0.84	18.5±1.69	0.010^*^
	6	Ability of delivering scientific, analytical concept s of course material	19.1±1.02	18.6±1.47	0.140
	7	Ability of scientific and logical answer to student questions	19.0±1.13	18.6±1.45	0.180
	8	Effectiveness of homework indicated by professor related to learning process to achievement of educational goals	19.2±0.91	18.5±1.35	0.020^*^
**Evaluation **	9	Routine assessment of student progress during the semester course and proposing method of improvement weakness and supporting strength point	18.2±1.47	18.8±1.01	0.030^*^
	10	Delivering the class lecture according to advanced scientific knowledge	19.1±1.08	18.7±1.14	0.150
** Professional and skillful communication**	11	Introducing the necessary, useful and advanced references	19.1±1.29	18.8±1.11	0.280
	12	Mutual respect of professor and students	19.6±0.62	19.3±1.17	0.110
** Class management skill **	13	Applying appropriate ethical, Islamic and professional consideration related to training duties	19.7±0.62	19.5±0.82	0.450
	14	Appropriate learning benefit and educational performance of student from offered coursework	19.0±0.71	18.7±1.29	0.550
**Total scores**			269.0±9.7	265.7±14.6	0.280

* Significant level: 0.05

The mean score of the male faculty members’ self-assessment was 269.3±8.4, while that of the female faculty was 268.0±13.1; however, the difference was not statistically significant (t=0.35, p=0.720). Additionally, the mean score of male and female faculty members’ evaluation by students was 266.2±15.2 and 262.5±12.3, respectively. Therefore, the difference was not statistically significant (t=0.74, p=0.46).   

## Discussion

Faculty members and students have different perceptions of which behaviors an ideal faculty member should possess. For instance, the faculty indicated that ideal profes­sors should help their students develop general learn­ing skills as well as an intrinsic interest in learning. On the other hand, the students emphasized that the ideal faculty should make educational materials, such as handouts, accessible to students and have socially appropriate behaviors. The faculty and students also disagreed about which behaviors professors should actually pos­sess (13).

The present study aimed to compare the evaluation of the faculty’s educational performance by students and the faculty’s self-assessment in School of Health and Nutrition, Shiraz University of Medical Sciences. According to the results, the total score of professors’ evaluation by undergraduate students was significantly lower than the faculty’s self- assessment. These results were supported by those obtained by Aghamolaei and Abedini (14), Goharian (15) and Sooki (16). In the research by Aghamolaei and Abedini, educational performance evaluation scores by undergraduate students were lower than the faculty’s self-assessment scores. In addition, Goharian and colleagues indicated that the educational performance evaluation scores by residency students were lower than the attending professors’ self-assessment scores (15). Sooki also compared the educational performance evaluation of the faculty members of the midwifery department by midwifery students and faculties (16). The midwifery professors’ self-assessments showed that they had higher scores and more positive points of view about their training performance compared to the students. Furthermore, Rafaee and Safi carried out a study to identify the factors related to the students’ evaluation of the educational performance of the professors of Arak University of Medical Sciences (17). The results indicated that the faculty members’ understanding of the quality and content of educational performance was different from the students’ viewpoints.

The findings of the present study demonstrated no statistically significant difference between the faculty’s self-assessment and graduate students’ viewpoints. The results were in contrast with those of the previous studies (9-12). This difference might be due to the fact that the previous studies were conducted on undergraduate students (14-17). Another explanation might be that undergraduate students are more dependent on their faculty, while graduate ones have a better understanding of the educational system and, consequently, their point of view is closer to the faculty’s perspective and expectation.

Also, the results of this study showed a weak correlation between the faculty’s self-assessment and their evaluation by students.  These findings are in the same line with those of the studies by Aghamolaei and Abedini (14), Vahidshahi et al. (18), and Miron (19). Vahidshahi et al. (18) studied the consistency of clinical professors’ training method and students' perspective in Sari Medical School. That study indicated a weak correlation between the students’ evaluation of the professors’ educational performance and the faculty’s self-assessment.

Similar to other studies (14, 20), the findings of the current study showed a statistically significant difference between the undergraduate students’ evaluation of the professors’ educational performance and the faculty’s self-assessment. In case no statistically significant difference was observed in this regard, it would imply that both parties realized their weaknesses and strengths. Nonetheless, a significant difference was found between the professors’ and undergraduate students’ evaluation scores in 12 out of 15 questions. If a statistically significant difference was found between evaluation of professors by undergraduate students and faculty’s self-assessment, it would imply that the faculty members and students had different views about a similar issue. The results indicated a statistically significant difference between the faculty’s self-assessment and evaluation of the faculty by graduate students in 3 out of 14 questions in the questionnaire. Thus, it seems that the faculty and graduate students had a similar understanding of similar questions.

The small number of graduate students compared to the large number of undergraduate students allows the former to have better communication with the faculty members and a better chance to understand the educational system. On the other hand, the small sample of the students in the graduate school could not lead to a reliable comparison and, as a result, further studies with larger sample sizes are required to be conducted on the issue. 

The findings of this study revealed no statistically significant difference between the professors’ self-assessment scores and the students’ evaluation scores based on their gender. These findings are compatible with those of the study by Aghamolaei and Abedini (14). However, Fleischman and Williams performed a study in Indiana St. College of USA and showed that female students’ evaluation of the faculty’s educational performance was more reasonable compared to the males (21).

One of the limitations of this study was the small number of the faculty members and the students who evaluated them. Thus, future studies with larger sample sizes should be conducted to better understand the differences between the faculty' self-assessment and their evaluation by students.

## Conclusion

Students and faculty members are two essential elements in evaluation of the educational performance. Therefore, having a view of their idea about teaching methods is of great importance. The results of the faculty’s self-assessment should be used to improve the quality of the course content and their teaching method. Consequently, professors should count on the students’ evaluation and improve their weaknesses. Overall, we conclude that mutual appropriate communication and analytical understanding of the faculty and students in the graduate school are more feasible compared to undergraduate school. According to the findings of this study, whether or not there is a correlation, similarity, or compatibility between the students' opinions about the faculty’s training performance and the faculty members’ beliefs, it is necessary to focus on needs and educational performance of the undergraduate students.
